# Therapeutic Potential of *Plantago ovata* Bioactive Extracts Obtained by Supercritical Fluid Extraction as Influenced by Temperature on Anti-Obesity, Anticancer, and Antimicrobial Activities

**DOI:** 10.3390/plants14121813

**Published:** 2025-06-12

**Authors:** Husam Qanash, Abdulrahman S. Bazaid, Naif K. Binsaleh, Amirah S. Alshammari, Reem Eltayeb

**Affiliations:** 1 Department of Medical Laboratory Science, College of Applied Medical Sciences, University of Ha’il, Hail 55476, Saudi Arabia; ar.bazaid@uoh.edu.sa (A.S.B.); n.binsaleh@uoh.edu.sa (N.K.B.); amerare_24@icloud.com (A.S.A.); re.ahmed@uoh.edu.sa (R.E.); 2 Medical and Diagnostic Research Center, University of Ha’il, Hail 55473, Saudi Arabia

**Keywords:** medicinal plants, supercritical fluid extraction, polyphenols, anticancer, anti-obesity, antimicrobial activity

## Abstract

*Plantago ovata* has been utilized as an effective natural remedy with minimal side effects, offering a promising alternative to synthetic pharmaceuticals. The supercritical fluid extraction (SFE) of *Plantago ovata* leaves yielded 0.417 g and 0.532 g at 40 °C and 80 °C, respectively. The 40 °C extract exhibited stronger antimicrobial activity, with minimum inhibitory concentrations (MICs) as low as 15.62 µg/mL and minimum bactericidal concentrations (MBCs) as low as 31.25 µg/mL against *Bacillus subtilis* and *Candida albicans*. In contrast, the 80 °C extract demonstrated reduced activity, with MICs and MBCs up to 250 and 500 µg/mL, respectively. The 40 °C extract also showed superior lipase inhibition (IC_50_ = 17.21 µg/mL) compared to the 80 °C extract (IC_50_ = 26.42 µg/mL), although orlistat remained the most potent (IC_50_ = 6.02 µg/mL). In addition, cytotoxicity assays revealed stronger effects of the 40 °C extract on Caco-2 colon cancer cells (IC_50_ = 109.47 µg/mL) compared to the 80 °C extract (IC_50_ = 174.81 µg/mL). These results suggest that the lower-temperature SFE of *P. ovata* yields extracts with enhanced antimicrobial, anti-obesity, and anticancer activities, supporting its potential for pharmaceutical and nutraceutical applications.

## 1. Introduction

Non-communicable disorders (NCDs) constitute a substantial worldwide public health challenge, leading to nearly 41 million adult fatalities annually [1]. Among these, obesity ranks as one of the most prevalent and preventable contributors, accounting for up to 5 million adult deaths each year, particularly in individuals with a body mass index (BMI) exceeding 25 kg/m^2^ [2]. Obesity significantly increases the risk of four chronic and life-threatening diseases, such as cardiovascular disease, diabetes mellitus (DM), stroke, and cancer, which are collectively responsible for nearly 4 million deaths annually [3]. According to the 2024 World Obesity Atlas (WOA), the number of overweight children aged 5 to 19 is expected to reach 750 million by 2035, with the highest burden anticipated in developing countries [4]. This trend suggests a dramatic rise in obesity-associated conditions in the near future, highlighting the urgent need for preventive and therapeutic strategies targeting this public health crisis.

Obesity is a complex chronic condition characterized by the excessive accumulation of white adipose tissue due to poor dietary habits, sedentary lifestyles, or underlying metabolic dysregulation [5]. The World Health Organization (WHO, 2018) classifies individuals as overweight or obese based on BMI, with global estimates indicating approximately 650 million obese and 1.9 billion overweight individuals [6]. Obesity predisposes individuals to a multitude of comorbidities, including cardiovascular diseases, diabetes mellitus, hepatic steatosis, and the chronic inflammation of adipose tissue and pancreatic islets [7,8,9]. Critically, obesity also plays a prominent role in the development and progression of various cancers [10,11]. Adiposity-driven inflammation, oxidative stress, insulin resistance, and altered adipokine secretion contribute to cancer pathogenesis by promoting cellular proliferation, angiogenesis, and immune evasion [12]. Colorectal cancer, in particular, has been strongly associated with obesity, with experimental evidence demonstrating that human colon epithelial cell lines (e.g., Caco-2) exposed to obesity-related metabolic stress exhibit increased DNA damage, resistance to apoptosis, and morphological alterations indicative of malignant transformation [13,14].

Although pharmacological approaches, such as appetite suppressants (e.g., phentermine), lipase inhibitors (e.g., orlistat), and bariatric procedures (e.g., gastric bypass surgery), are widely used to manage obesity [15,16], these methods often have adverse effects and limited long-term success. As a result, there is growing interest in safer plant-derived therapies that offer multifaceted health benefits. Plant-based interventions, especially those rich in bioactive compounds, are increasingly recognized for their anti-obesity, anti-inflammatory, and metabolic regulatory properties [17,18]. These natural compounds modulate obesity by inhibiting adipocyte differentiation, suppressing adipogenesis, regulating lipid metabolism, and reducing triacylglycerol accumulation and lipase activity [19,20]. Moreover, diets high in refined carbohydrates and animal proteins and low in fiber further aggravate the obesity burden [21,22].

Among the most promising medicinal plants is *P. ovata* (psyllium), which belongs to the Plantaginaceae family and includes over 250 species, primarily found in temperate regions [23,24]. The seeds and husks of *P. ovata* are traditionally used for their laxative, anti-inflammatory, and cholesterol-lowering properties, and have been cultivated across Europe, India, and Pakistan for centuries [25]. These benefits are attributed to the plant’s high content of indigestible fiber, mucilage, and volatile oils, which promote gastrointestinal health, regulate bowel function, and reduce blood cholesterol and glucose levels [25,26]. The seed husks also support gut microbiota through endogenous short-chain fatty acids, making *P. ovata* effective in managing gastrointestinal disorders such as colitis [27].

Beyond its metabolic and digestive benefits, *P. ovata* has exhibited notable antimicrobial activity. This is particularly significant in light of the global rise in multi-drug-resistant (MDR) bacteria, especially in regions where antibiotic misuse is prevalent [28]. Pathogens such as *Bacillus cereus*, a Gram-positive spore-forming bacterium, and *Pseudomonas aeruginosa*, a Gram-negative bacterium, are commonly implicated in foodborne and hospital-acquired infections and are increasingly resistant to conventional antibiotics [29,30]. Bioactive constituents of *P. ovata*, including arabinose, D-xylosan, D-galactose, and D-galacturonic acid, have shown antimicrobial activity against a range of bacterial pathogens, such as *Staphylococcus aureus*, *Streptococcus pyogenes*, and *Bordetella bronchiseptica,* positioning this plant as a viable candidate for natural antibacterial therapy [31].

To preserve and concentrate the plant’s pharmacologically active constituents, advanced green technologies, such as supercritical fluid extraction (SFE), are increasingly employed in this study. SFE, particularly using supercritical carbon dioxide (SC-CO_2_), is regarded as an environmentally friendly, non-toxic, and efficient extraction method capable of preserving thermolabile compounds under mild temperature and pressure conditions [32]. SC-CO_2_ combines the solvating power of liquids with the diffusion properties of gases, enabling selective and high-purity extraction without solvent residues [33]. Its efficiency largely depends on its driving force that shows the ability to dissolve target solutes through favorable solvent–solute interactions [34,35].

To investigate the therapeutic potential of *P. ovata* extracts, this study employed SFE at two distinct temperatures (40 °C and 80 °C), with a focus on evaluating their anti-obesity, anticancer, and antimicrobial activities. The findings underscore the potential of *P. ovata* as a natural source of multifunctional bioactive compounds for the prevention and treatment of non-communicable diseases, such as obesity and cancer, as well as communicable diseases caused by drug-resistant bacterial pathogens.

## 2. Results and Discussion

### 2.1. Supercritical Fluid Extraction (SFE) of Leaves

**Figure 1:**
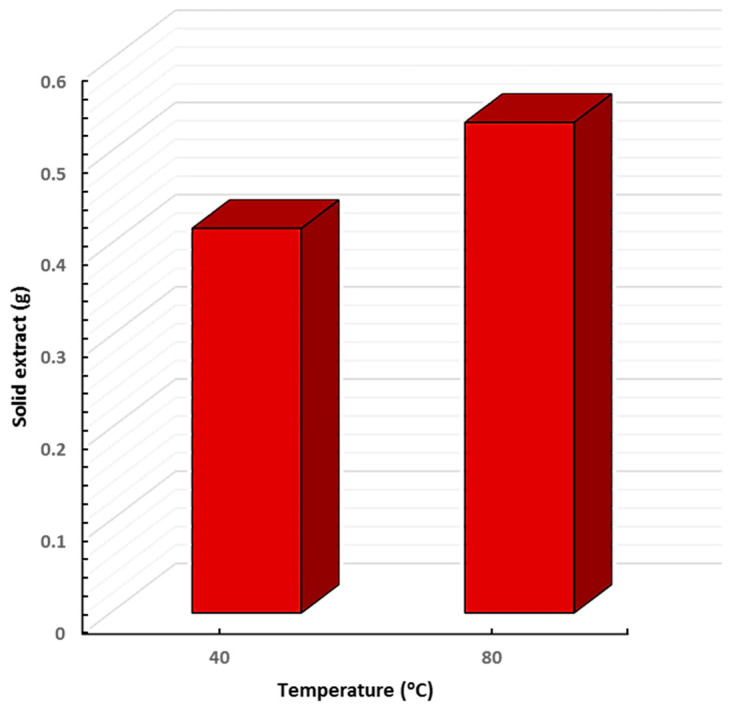
Solid extract content of *P. ovata* leaves at two temperatures.

Fresh *P. ovata* leaves were collected from private nurseries and promptly transported to the laboratory to preserve their phytochemical integrity. These leaves are recognized for their rich content of therapeutic constituents, as reported in multiple phytochemical studies. To optimize the recovery of these bioactive compounds, the extraction process was carried out under precisely controlled conditions. SFE was performed at two different temperatures (40 °C and 80 °C) under a constant pressure of 4000 psi. Each extraction consisted of a 15 min static phase followed by a 45 min dynamic phase. The use of two temperature settings aimed to compare both the yield and biological efficacy of the resulting extracts. The solid extract yield obtained from *P. ovata* leaves was 0.417 g at 40 °C and 0.532 g at 80 °C from 15 g of dried *P. ovata* leaf powder, corresponding to 2.78% and 3.54%, respectively, based on the dry weight. These results indicate that higher extraction temperatures enhance the recovery of bioactive compounds (Figure 1).

Comparable studies support the significance of temperature and method optimization in extraction yield. Maria et al. (2024) [36] reported that dried *P. major* L. seeds contained approximately 10% (*w*/*w*) moisture and 18.4% (*w*/*w*) total extractable matter. A modified extraction system incorporating Teflon^®^ beads and internal glass baffles significantly enhanced the yield by increasing mechanical abrasion between seeds and surrounding particles. Conventional extraction yielded 12.84% extract, corresponding to 69.8% of total seed mucilage, while the modified method achieved a higher yield of 14.17%, demonstrating improved efficiency. Assi et al. (2024) [37] evaluated psyllium mucilage extraction from *P. ovata* Forsk using various independent variables, including temperature (50–80 °C), extraction duration (60–120 min), water-to-seed ratio (50:1 to 100:1), and water pH (4–10). Dependent variables included extraction yield, swelling capacity, emulsifying capacity, and emulsion stability. The optimal extraction conditions (79.99 °C for 60.02 min, with a water-to-seed ratio of 99.99:1 and pH 7.38) resulted in an extraction yield of 29.54%, swelling capacity of 25.47 mL/g, emulsifying capacity of 68.39%, and emulsion stability of 76.61%. Similarly, Campos et al. (2016) [38] found that the highest extraction yield of chia mucilage was achieved at 80 °C. Bazezew et al. (2022) [39] investigated mucilage extraction from *Ximenia americana* seeds over a wide range of temperatures (15–110 °C), extraction times (0.5–6 h), and water-to-seed ratios (10:1 to 100:1). Golalikhani et al. (2014) [40] reported that mucilage yield from *X. americana* peaked at 69 °C before declining, while *Descurainia sophia* (L.) Webb ex Prantl seed mucilage exhibited maximum yield at 94.3 °C, followed by a decrease attributed to the thermal degradation of polysaccharide structures. The thermal degradation of phenolic compounds in *P. ovata* at 80 °C during SFE is unlikely to be significant, as this temperature falls within a safe range for preserving phenolic integrity. However, prolonged exposure, even at this temperature, or potential synergistic effects with other processing steps (e.g., drying or storage) may contribute to gradual degradation and should, therefore, be carefully monitored.

### 2.2. HPLC Analysis of P. ovata SFEs

**Figure 2:**
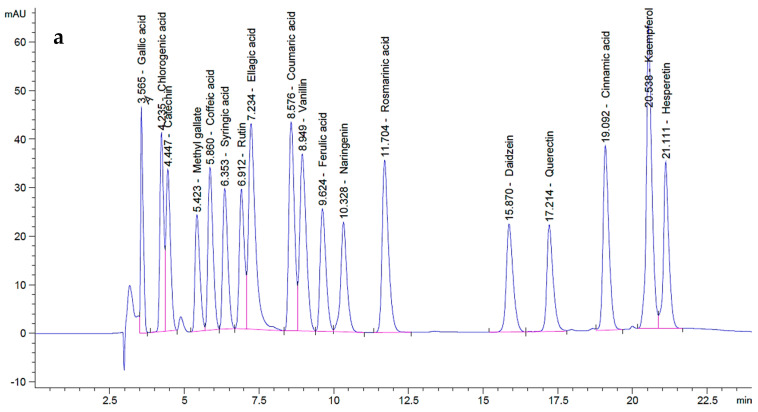

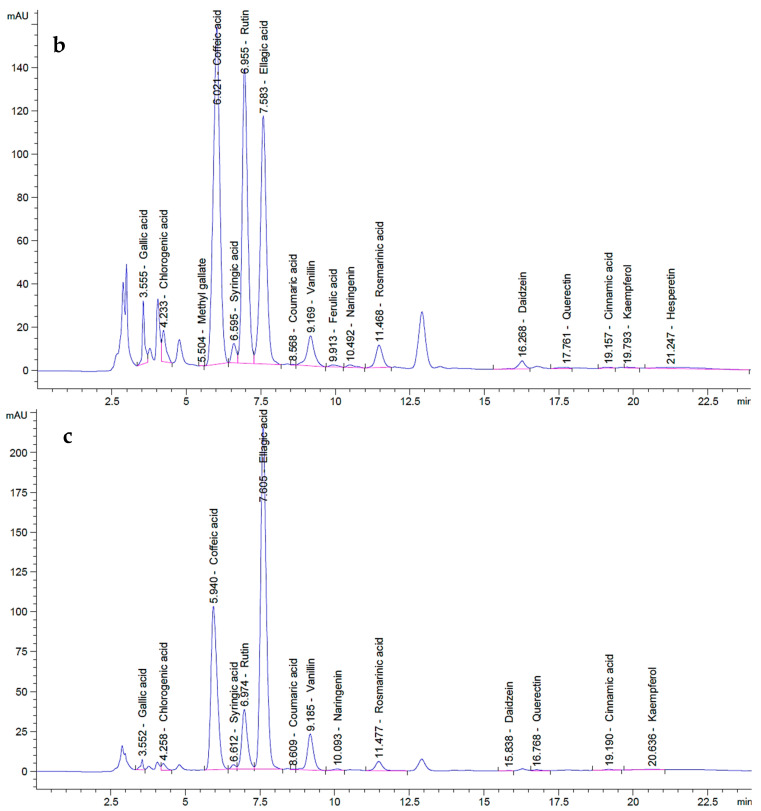
HPLC analysis for *P. ovata* leaf mucilage: (**a**) standard polyphenols, (**b**) SFE 40 °C, and (**c**) SFE 80 °C.

*P. ovata* leaf extracts obtained via SFE at 40 °C and 80 °C were analyzed by HPLC in comparison with standard polyphenolic compounds and revealed a total of 18 distinct components across the two extracts, each present at varying concentrations (Figure 2). In the chromatogram of the polyphenol standards (Figure 2a), each compound exhibited distinct retention times and peak areas, serving as references for compound identification in the extracts. The chromatograms of the *P. ovata* leaf extracts at 40 °C (Figure 2b) and 80 °C (Figure 2c) displayed multiple peaks, corresponding to various polyphenols, with differences in both retention time and peak area.

The quantitative analysis of the indigenous components in both extracts was documented (Table 1). In addition, the total polyphenol contents of the extracts obtained at 40 °C and 80 °C are also reported (Table 1). The 40 °C extract contained higher concentrations of several bioactive polyphenols than the 80 °C extract. For instance, gallic acid was present at 11.69 µg/mL in the 40 °C extract compared to 2.77 µg/mL in the 80 °C extract, while the standard concentration was 20 µg/mL. Similarly, chlorogenic acid was quantified at 18.81 µg/mL (40 °C) and 5.54 µg/mL (80 °C) versus 50 µg/mL in the standard. Other compounds detected at higher levels in the 40 °C extract included methyl gallate (0.06 µg/mL; absent in 80 °C), caffeic acid (123 µg/mL vs. 76.51 µg/mL), syringic acid (5.65 µg/mL vs. 1.70 µg/mL), rutin (264.90 µg/mL vs. 75.80 µg/mL), ferulic acid (1.01 µg/mL; absent in 80 °C), naringenin (2.07 µg/mL vs. 1.35 µg/mL), rosmarinic acid (16.10 µg/mL vs. 9.83 µg/mL), daidzein (3.43 µg/mL vs. 0.11 µg/mL), quercetin (1.82 µg/mL vs. 1.31 µg/mL), and hesperetin (3.04 µg/mL; absent in 80 °C). Notably, catechin was undetectable in both extracts but was present in the standard at 75 µg/mL. Conversely, some polyphenols were more abundant in the 80 °C extract, including ellagic acid (290.57 µg/mL vs. 168.62 µg/mL in 40 °C), coumaric acid (0.13 µg/mL vs. 0.05 µg/mL), vanillin (11.92 µg/mL vs. 8.69 µg/mL), and kaempferol (0.31 µg/mL vs. 0.18 µg/mL). Cinnamic acid was found in equal concentrations (0.19 µg/mL) in both extracts.

Overall, the SFE extract obtained at 40 °C was richer in key polyphenols with well-documented anti-obesity and antimicrobial properties, such as gallic acid, chlorogenic acid, caffeic acid, rutin, rosmarinic acid, and quercetin, compared to the extract obtained at 80 °C. These differences suggest that extraction at 40 °C more effectively preserves thermolabile compounds, thereby enhancing the therapeutic potential of the extract. In contrast, certain compounds that were more abundant in the 80 °C extract (e.g., ellagic acid and coumaric acid) are known to exhibit relatively weaker anti-obesity and antimicrobial effects, which may account for the reduced bioactivity observed in the biological assays at this temperature.

These findings are supported by Pratik et al. (2015) [41], who reported high levels of gallic acid and rutin in *P. ovata* extracts, especially in callus cultures, attributed to the increased expression of the phenylalanine ammonia-lyase (PAL) gene involved in polyphenol biosynthesis. Polyphenols are secondary metabolites abundantly found in higher plants, including vegetables [42], fruits [43,44], spices [45], grains [46], legumes, and nuts [47]. These compounds exhibit a broad range of therapeutic effects, including antioxidants, antimicrobial, anticancer, and anti-inflammatory activities. Structurally, polyphenols are characterized by one or more hydroxylated aromatic rings, and they are broadly classified into flavonoids, phenolic acids, tannins, stilbenes, and lignans [48].

### 2.3. Assay of Antimicrobial Activity

**Figure 3:**
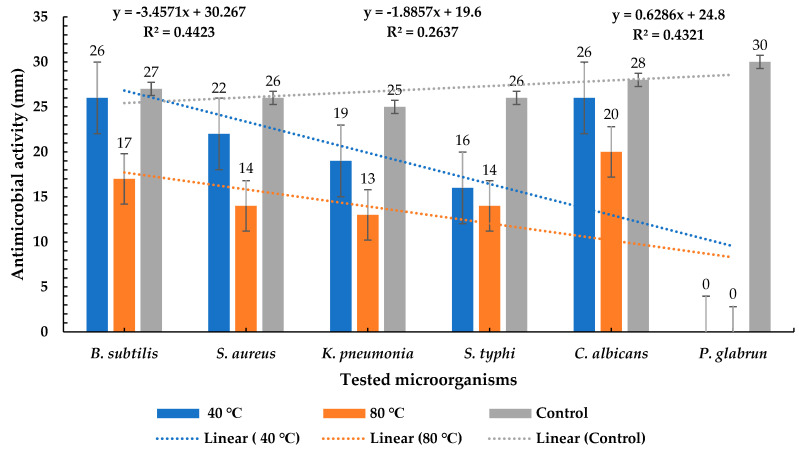
Antimicrobial activity of *P. ovata* extract at two temperatures.

**Figure 4:**
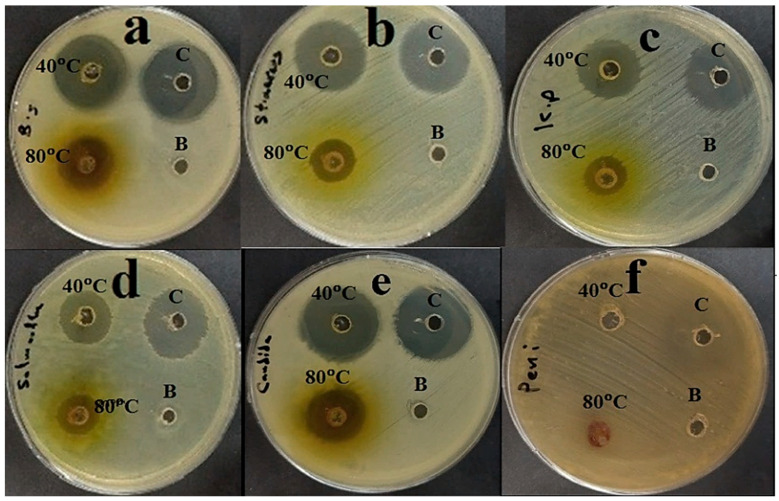
Antimicrobial activity of *P. ovata* extract at 40 °C and 80 °C compared to positive antibiotic control (c) and blank (B), as well as negative control against (**a**) *B. subtilis*, (**b**) *S. aureus*, (**c**) *K. pneumonia*, (**d**) *S. typhi*, (**e**) *C. albicans*, and (**f**) *P. glabrum*.

*P. ovata* extracts obtained at 40 °C and 80 °C demonstrated antimicrobial activity against a broad range of microorganisms, including *B. subtilis*, *S. aureus*, *K. pneumoniae*, *S. typhi*, and *C. albicans*. However, no inhibitory effect was observed against *P. glabrum*, which exhibited dense fungal growth. These findings suggest that the *P. ovata* extracts possess broad-spectrum antimicrobial activity, with the 40 °C extract exhibiting superior efficacy compared to the 80 °C extract (Figure 3). Quantitatively, the 40 °C extract inhibited *B. subtilis* with a zone of inhibition of 26 ± 1 mm, while the 80 °C extract yielded 17 ± 1 mm, compared to 27 ± 1 mm for the gentamycin control (Figure 4a). In the case of *S. aureus*, the inhibition zones were 22 ± 1 mm and 14 ± 1 mm for the 40 °C and 80 °C extracts, respectively, versus 26 ± 1 mm for the control (Figure 4b). Against *K. pneumoniae*, inhibition zones measured 19 ± 1 mm for the 40 °C extract and 13 ± 1 mm for the 80 °C extract, with the control yielding 25 ± 1 mm (Figure 4c). The inhibition of *S. typhi* was 16 ± 1 mm and 14 ± 2 mm for the 40 °C and 80 °C extracts, respectively, compared to 26 ± 1 mm for the control (Figure 4d). *C. albicans* was inhibited at 26 ± 2 mm by the 40 °C extract and 20 ± 1 mm by the 80 °C extract, while fluconazole (used as a positive control) showed an inhibition zone of 28 ± 1 mm (Figure 4e). In contrast, *P. glabrum* showed no inhibition from either extract, whereas the control exhibited a clear inhibition zone of 30 ± 1 mm (Figure 4f). These results indicate that *P. ovata* extracts were generally more effective against Gram-positive than Gram-negative bacteria, and the antifungal activity against *C. albicans* closely mirrored that observed against Gram-positive organisms. The reduced activity of the 80 °C extract suggests that higher temperatures may degrade thermolabile bioactive compounds.

These findings are consistent with those of Saoussen et al. (2020) [49], who reported that essential oils from the aerial parts of *P. afra* L. exhibited broad-spectrum antimicrobial activity against both Gram-negative (*Escherichia coli*, *Salmonella enterica*, *Pseudomonas aeruginosa*) and Gram-positive bacteria (*S. aureus* and *B. subtilis*), as well as *C. albicans*. Similarly, Ravi et al. (2018) [50] demonstrated that *P. ovata* Forsk exhibited antibacterial activity against *Fusobacterium nucleatum*, *Aggregatibacter actinomycetemcomitans*, *Porphyromonas gingivalis*, and *Prevotella intermedia*, with MICs of 12.5 μL/mL, 50 μL/mL, 0.8 μL/mL, and 0.4 μL/mL, respectively. Among these, *A. actinomycetemcomitans* was the most resistant, whereas *P. intermedia* was the most sensitive.

### 2.4. Determination of MICs and MBCs of P. ovata Extracts

**Figure 5:**
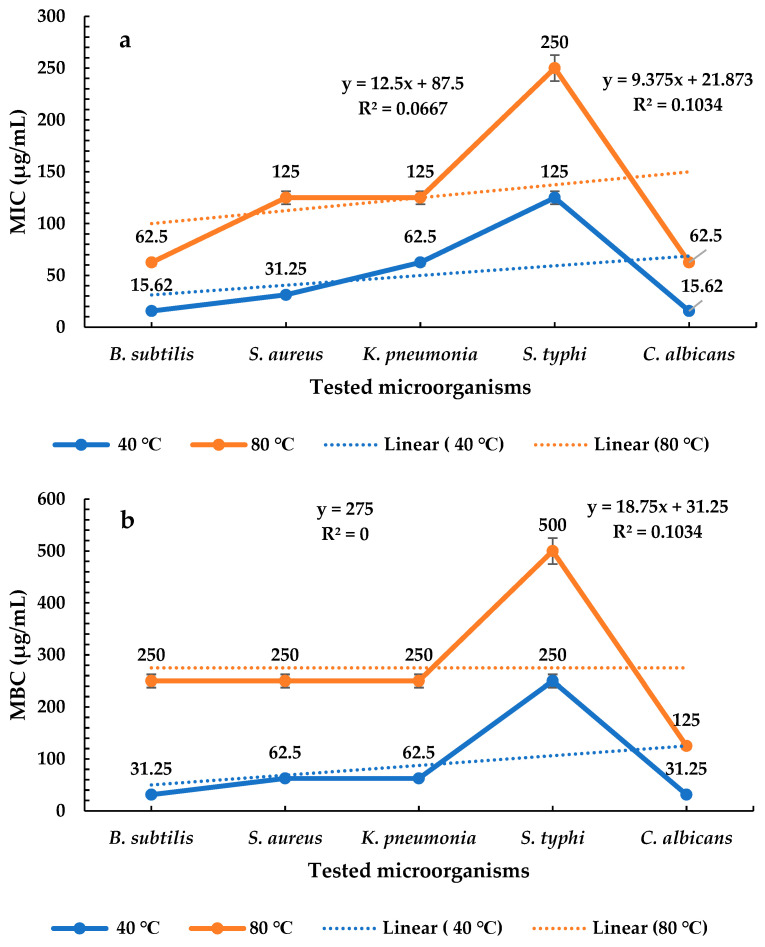
Evaluation of *P. ovata* extract (SFE 40 °C and SFE 80 °C): (**a**) MICs and (**b**) MBCs against *B. subtilis*, *S. aureus*, *K. pneumonia*, *S. typhi*, and *C. albicans*.

*P. ovata* extracts obtained at 40 °C and 80 °C exhibited varying MICs and MBCs against a wide selection of both bacterial and fungal pathogens. The extract obtained at 40 °C showed the lowest MIC of 15.62 µg/mL against *Bacillus subtilis* (Gram-positive) and *Candida albicans*, followed by 31.25 µg/mL against *Staphylococcus aureus* (Gram-positive), 62.50 µg/mL against *Klebsiella pneumoniae*, and 125 µg/mL against *Salmonella typhi* (both Gram-negative). These results suggest that the 40 °C extract was more effective against Gram-positive bacteria and yeast than against Gram-negative bacteria. In contrast, the 80 °C extract showed its lowest MIC of 62.50 µg/mL against *B. subtilis* and *C. albicans*, followed by 125 µg/mL against both *S. aureus* and *K. pneumoniae*, and 250 µg/mL against *S. typhi* (Figure 5a). These data indicate that, while both extracts exhibited broader efficacy against Gram-positive bacteria and yeast, the extract prepared at 40 °C consistently showed superior antimicrobial potency. Among Gram-positive bacteria, *S. aureus* showed reduced sensitivity compared to *B. subtilis*, whereas, among Gram-negative bacteria, *S. typhi* was less susceptible compared to *K. pneumoniae*. Overall, the 40 °C extract demonstrated stronger microbial inhibition than the 80 °C extract across all tested organisms, particularly against Gram-positive bacterial and fungal pathogens. Regarding MBCs, the 40 °C extract showed the lowest value of 31.25 µg/mL against *C. albicans*, followed by 31.25 µg/mL against *B. subtilis*, 62.50 µg/mL against *S. aureus* and *K. pneumoniae*, and 250 µg/mL against *S. typhi*. These findings confirm the higher efficacy of the 40 °C extract, particularly against yeast (Figure 5b). The 80 °C extract exhibited a minimum MBC of 125 µg/mL against *C. albicans*, followed by 250 µg/mL against *B. subtilis*, *S. aureus*, and *K. pneumoniae*, and 500 µg/mL against *S. typhi*, suggesting diminished activity at a higher extraction temperature.

These findings align with the observations of Saoussen et al. (2020) [49], who reported that essential oil from *P. afra* L. exhibited MICs and MBCs ranging from 312.5 to 1250 µg/mL against Gram-positive and Gram-negative bacteria, as well as molds. Notably, the essential oil demonstrated greater inhibitory activity against Gram-positive than Gram-negative bacteria, with *S. aureus* and *B. subtilis* showing MICs and MBCs of 625 µg/mL. Moreover, the essential oil was more effective against *C. albicans*, showing a lower MIC and MBC (312.5 µg/mL) compared to bacterial isolates. In a related study, Garcia-Salinas et al. (2018) [51] found that the essential oil of *P. afra*, rich in phenyl derivatives such as thymol, exhibited broad-spectrum antimicrobial activity. Thymol demonstrated the highest inhibitory effect, particularly against *Escherichia coli* and *S. aureus*, with MIC values as low as 200 µg/mL.

### 2.5. Assay of Pancreatic Lipase Inhibition

**Figure 6:**
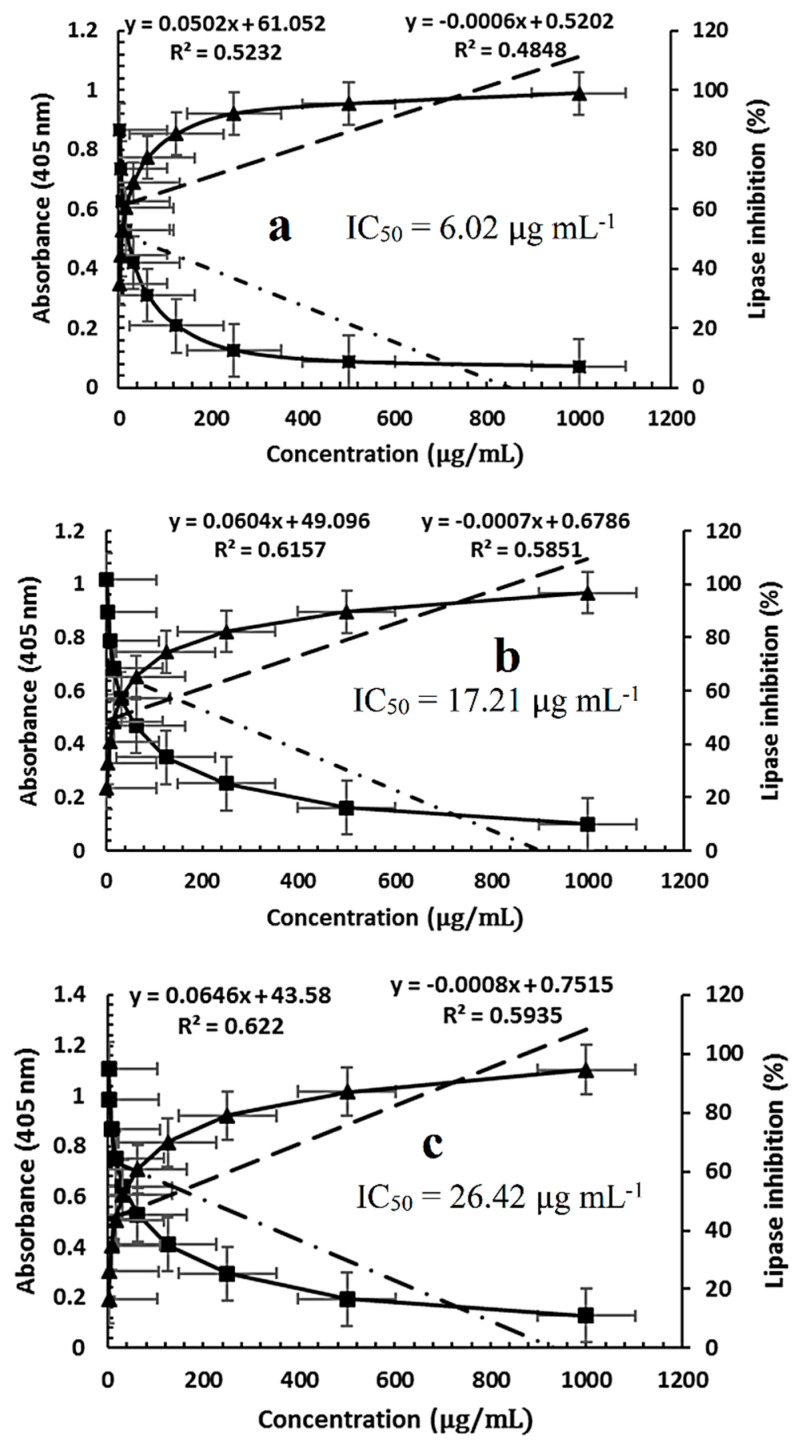
Lipase inhibitory activity: (**a**) standard orlistat, (**b**) SFE 40 °C, and (**c**) SFE 80 °C.

Different concentrations of *P. ovata* SFE obtained at 40 °C and 80 °C were evaluated for their lipase inhibitory activity in comparison with the standard anti-obesity drug orlistat. Absorbance was measured at 405 nm in triplicate, and the percentage of lipase inhibition was calculated along with SD and SE. Orlistat at its highest concentration (1000 μg/mL) showed the greatest lipase inhibition at 99% with an SD of 0.009 and SE of 0.003. Inhibitory activity decreased progressively with lower concentrations, showing inhibition rates of 95.4%, 92.1%, 85.3%, 77.3%, 68.8%, 60.4%, 53.1%, 44.6%, and 34.9% at concentrations of 500, 250, 125, 62.50, 31.25, 15.63, 7.81, 3.91, and 1.95 μg/mL, respectively. Complete loss of inhibition was observed at the lowest tested concentration. The IC_50_ for orlistat was determined to be 6.02 μg/mL (Figure 6a). The SFE extract at 40 °C demonstrated a maximum lipase inhibition of 96.8% at 1000 μg/mL with an SD of 0.007 and SE of 0.002. A concentration-dependent decline in inhibitory activity was observed, with inhibition percentages of 89.7%, 82.3%, 74.5%, 65.4%, 57.4%, 48.5%, 40.9%, 32.7%, and 23.5% across the same concentration range. The IC_50_ for the 40 °C extract was calculated as 17.21 μg/mL (Figure 6b). Similarly, the SFE extract at 80 °C exhibited a maximum lipase inhibition of 94.6% at 1000 μg/mL, with SD and SE values of 0.005 and 0.002, respectively. Inhibition percentages decreased with lower concentrations to 87.1%, 79.0%, 69.9%, 60.8%, 52.3%, 43.5%, 34.9%, 26.0%, and 16.7%, with an IC_50_ of 26.42 μg/mL (Figure 6c). Collectively, these findings demonstrate that both SFE extracts exerted dose-dependent lipase inhibitory activity, although the extract obtained at 40 °C was more potent than that extracted at 80 °C. Nevertheless, orlistat exhibited the highest inhibitory efficiency among all tested samples. The lower IC_50_ of the 40 °C extract suggests that extraction at a lower temperature may better preserve thermolabile lipase inhibitory constituents.

These results are consistent with findings by Ali and Narjes (2023) [52], who reported significant anti-obesity effects of *P. ovata*, particularly due to its ability to inhibit pancreatic lipase. Their study showed that oral consumption of 20 g of *P. ovata* seeds before or after meals for three days significantly reduced appetite within one hour and, consequently, lowered fat intake. Similarly, Hana et al. (2020) [53] described obesity as a metabolic disorder associated with severe non-communicable diseases such as diabetes mellitus, cardiovascular disease, and atherosclerosis. Contemporary anti-obesity strategies increasingly target the reduction of fat absorption by inhibiting pancreatic lipase, preferably through natural inhibitors with minimal side effects. Moreover, natural lipase inhibitors are derived from microorganisms and medicinal plants. For instance, lipstatin and its saturated derivative orlistat, isolated from *Actinobacteria* and *Streptomyces toxicaricini*, are among the most effective microbial inhibitors of pancreatic lipase. Although synthetic compounds have shown potent anti-obesity effects, they are often accompanied by adverse effects ranging from mild to severe [54,55,56]. In contrast, plant-derived lipase inhibitors, including polysaccharides [57], polyphenols [58], terpene trilactones [59], flavonoids [60], alkaloids [61], saponins [62], and carotenoids [63], offer safer and sustainable alternatives. The presence of these compound classes in *P. ovata* likely contributes to its strong lipase inhibitory and anti-obesity potential. Generally, the extract obtained at 80 °C exhibited lower activity across all biological assays, which may be attributed to the degradation of active compounds at elevated temperatures.

### 2.6. Cytotoxicity Test

**Figure 7:**
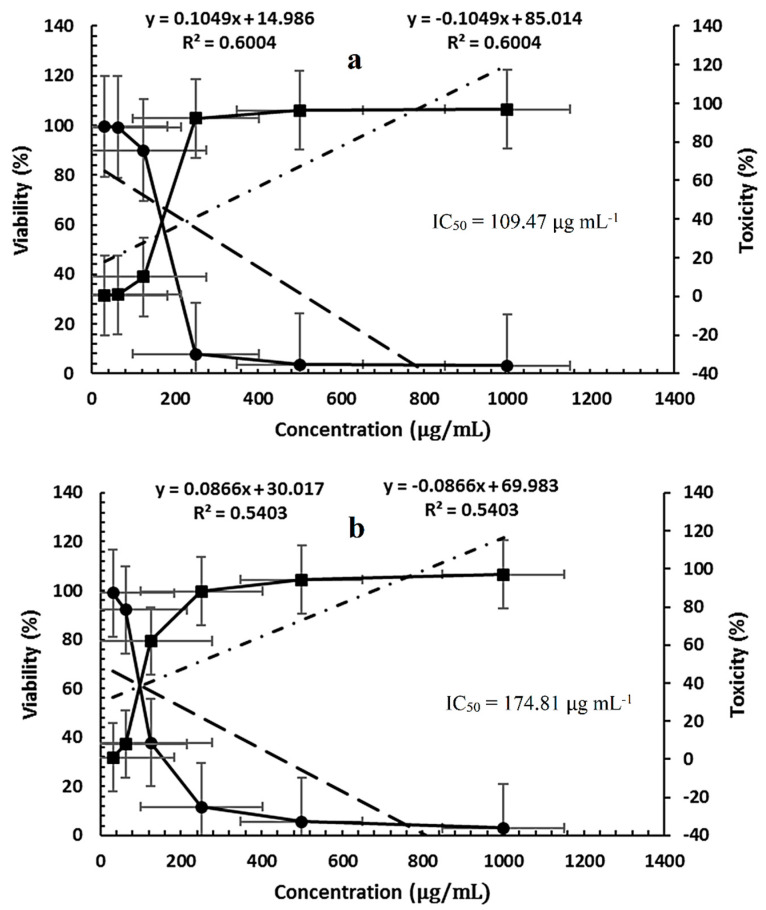
Cytotoxicity and viability of *P. ovata* leaves on Caco2 cell line: (**a**) SFE 40 °C and (**b**) SFE 80 °C.

**Figure 8:**
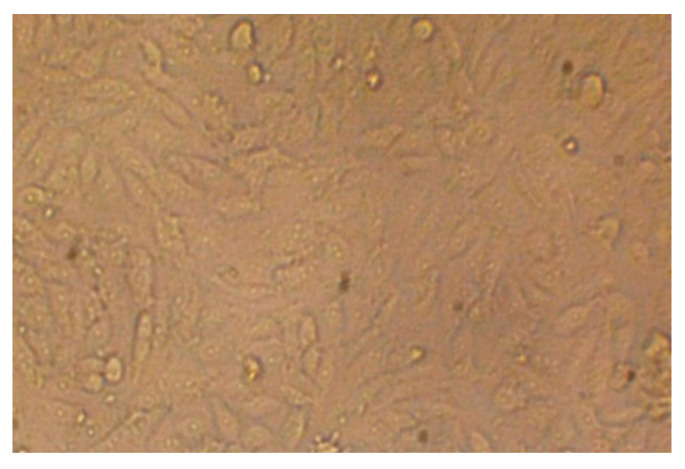
Untreated Caco2 cell line in colon tissues as positive control.

**Figure 9:**
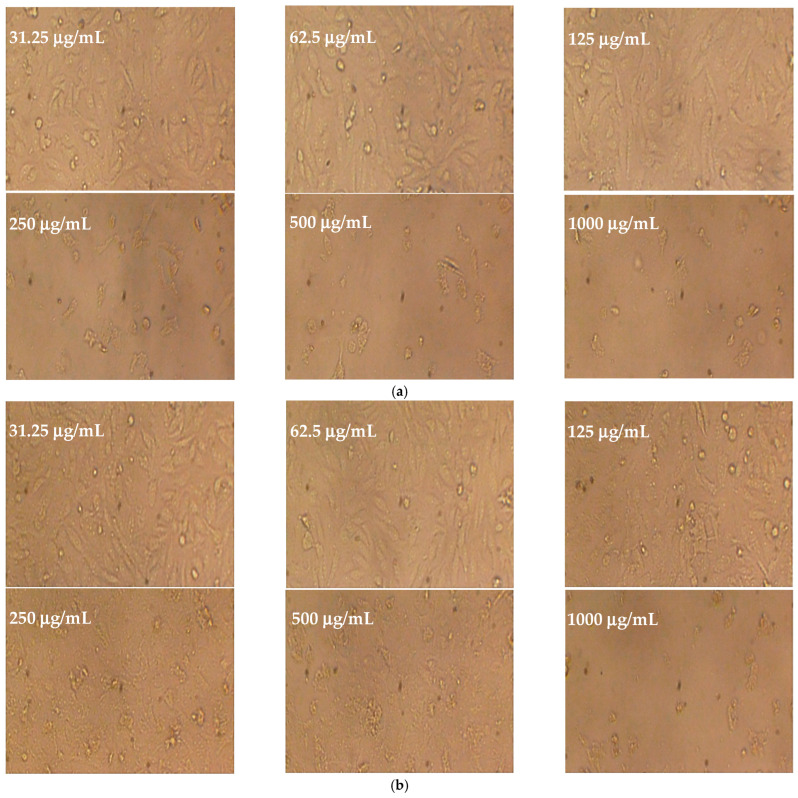
(**a**) Effect of different concentrations of *P. ovata* leaves to determine cytotoxicity and viability at SFE 40 °C. (**b**) Effect of different concentrations of *P. ovata* leaves to determine cytotoxicity and viability at SFE 80 °C.

Different concentrations of *P. ovata* leaf extracts obtained via SFE at 40 °C and 80 °C were evaluated for cytotoxicity and cell viability against the human colon epithelial cell line (Caco-2), with untreated cell line used as a control (Figure 7a,b, Figure 8 and Figure 9a,b). The SFE 40 °C extract demonstrated greater cytotoxicity toward Caco-2 cells than the SFE 80 °C extract, with IC_50_ values of 109.47 ± 1.01 μg/mL and 174.81 ± 1.5 μg/mL, respectively. At the highest tested concentration (1000 μg/mL), the SFE 40 °C extract caused 97.02% cytotoxicity with 2.971% viability, while the SFE 80 °C extract induced 96.86% cytotoxicity with 3.133% viability. Although toxicity decreased and viability increased with lower extract concentrations, SFE 80 °C consistently exhibited lower cytotoxicity and higher cell viability compared to SFE 40 °C. These results suggest a concentration-dependent cytotoxic effect and indicate that the lower extraction temperature retains more bioactive cytotoxic compounds. Although not tabulated, additional data indicated that the normal human fetal lung fibroblasts (WI38 cell line) were inhibited by the SFE 40 °C and 80 °C extracts, with IC_50_ values of 218.25 ± 1.33 μg/mL and 292.66 ± 2.33 μg/mL, respectively.

The observed cytotoxicity aligns with earlier findings by Marina et al. (2003) [64], who reported that methanolic extracts of several *Plantago* species exhibited cytotoxic effects against human cancer cell lines, including mammary adenocarcinoma (TK-10, MCF-7) and melanoma (UACC-62). Among the tested species, *P. bellardii* extract showed the highest activity against the TK-10 cell line (GI_50_ = 86 μg/mL), while methanolic extracts of *P. coronopus* and *P. bellardii* exhibited the greatest cytotoxicity against MCF-7 and UACC-62 cells, with GI_50_ values of 32 and 34 μg/mL, respectively. Total growth inhibition (TGI) of MCF-7 cells was observed with extracts from *P. bellardii*, *P. coronopus*, *P. lanceolata*, *P. major*, and *P. serraria* at TGI values of 87, 74, 99, 97, and 120 μg/mL, respectively. Likewise, the complete inhibition of UACC-62 cell growth was achieved with extracts of *P. afra*, *P. bellardii*, *P. coronopus*, *P. lagopus*, *P. major*, and *P. serraria*, with TGI values of 238, 84, 89, 244, 112, and 118 μg/mL, respectively. In contrast, none of the extracts tested induced complete growth inhibition in TK-10 cells. Furthermore, extracts of *P. coronopus*, *P. lanceolata*, *P. major*, and *P. serraria* achieved a net 50% cell killing rate on MCF-7 cells at concentrations of 169, 212, 207, and 274 μg/mL, respectively. On UACC-62 cells, *P. coronopus* and *P. major* extracts exhibited 50% killing rates at 198 and 247 μg/mL, respectively. Luteolin-7-O-glucoside isolated from *P. lagopus*, along with its aglycone luteolin, demonstrated notable cytotoxicity against all three cancer cell lines tested. The glucoside showed the highest activity against MCF-7 (GI_50_ = 40 μg/mL), whereas luteolin exhibited strongest effects against the melanoma UACC-62 line (GI_50_ = 10 μg/mL).

Collectively, these data suggest that the cytotoxicity of *P. ovata* SFE, particularly at 40 °C, may be attributed to the preservation of flavonoid compounds with anticancer properties, thus offering promising applications in colon cancer therapy. However, further studies are warranted to isolate and characterize the individual active constituents and elucidate their specific mechanisms of action.

## 3. Materials and Methods

### 3.1. Plant Materials, Chemical Reagents, and Cell Line Used

Fresh leaves of *P. ovata* were collected from private nurseries in Jazan City, Saudi Arabia. The plant materials were authenticated and documented by the Medical and Diagnostic Research Center, University of Ha’il, Ha’il, Saudi Arabia. Trifluoroacetic acid (Catalog No. TCI-T0431) and p-nitrophenyl butyrate (Catalog No. N2969) were purchased from Spectrum Chemical, Gardena, California, USA. Potassium phosphate buffer (Catalog No. P8584), lipase (Catalog No. A6255), and orlistat (Catalog No. O4139) were obtained from Sigma-Aldrich (St. Louis, MO, USA). The colon epithelial cell line (Caco-2) and normal human fetal lung fibroblasts (WI38 cell line) were kindly provided by VACSERA, Dokki, Giza Governorate, Egypt.

### 3.2. Supercritical Fluid Extraction (SFE) of Leaves

*P. ovata* leaves were freeze-dried using a benchtop freeze-drying apparatus (Benchtop, Labconco Corporation, Kansas City, MO, USA). The material was subjected to a temperature of −50 °C and a pressure reduced to a few millibars to facilitate sublimation, allowing the frozen water content to transition directly from the solid to the gaseous phase. A chilled condensation chamber and condenser plates offered a surface upon which the water vapor could undergo re-solidification. The dried leaves were then ground using a commercial blender (Blender 8011ES Model HGB2WTS3, 400 W, Scientific Laboratory Supplies Ltd., Nottingham, UK), followed by sieving through a metal mesh with 0.5 mm pore size. Fifteen (g) of dried *P. ovata* leaf powder was extracted using a supercritical carbon dioxide (SC-CO_2_) extraction system (SEPARECO, Boldon, Tyne & Wear, UK). The extraction was carried out with 16% (*w*/*w*) ethanol as a co-solvent, at a CO_2_ flow rate of 20 g/min, at temperatures of 40 °C and 80 °C, and at a constant pressure of 100 bar [65,66]. The resulting supercritical extract was weighted (g).

### 3.3. HPLC Analysis of P. ovata SFEs

The mucilage extract of *P. ovata* leaves was analyzed using high-performance liquid chromatography (HPLC) on an Agilent 1260 Infinity series system. Separation was achieved using a Zorbax Eclipse Plus C8 column (4.6 mm × 250 mm, 5 μm particle size) maintained at 40 °C. The mobile phase consisted of water (solvent A) and acetonitrile containing 0.05% trifluoroacetic acid (solvent B), delivered at a flow rate of 0.9 mL/min. A linear gradient elution program was employed beginning with 82% A at 0 min, maintained until 1 min, followed by a gradual reduction to 75% A from 1 to 11 min, then to 60% A from 11 to 18 min. The mobile phase composition was then returned to 82% A from 18 to 22 min and held constant until 24 min. Detection was carried out using a multi-wavelength detector set at 280 nm, and a 5 μL volume of each test sample was injected for analysis [67,68]. The total phenolic content (TPC) of *P. ovata* leaf powder was determined using the Folin–Ciocalteu reagent, following the method described by Anjum et al. (2017) [69]. Absorbance was measured at 725 nm using a Hitachi U-2900 spectrophotometer (Hitachi, Minato-ku, Tokyo, Japan), with the corresponding extraction solvent used as the blank. Results were expressed as milligrams of gallic acid equivalents per gram of *P. ovata* leaf powder (mg GAE/g).

### 3.4. Assessment of Antimicrobial Potential

The antimicrobial activity of the extracts was evaluated in vitro using the agar well diffusion method, as described by Qanash et al. (2025) [70]. Bacterial strains, including Gram-positive bacteria (*Bacillus subtilis* ATCC 6633 and *Staphylococcus aureus* ATCC 6538) and Gram-negative bacteria (*Klebsiella pneumoniae* ATCC 13883 and *Salmonella typhi* ATCC 6539), were cultured on Nutrient Agar (BD DIFCO, Beirut, Lebanon). Fungal strains, including the yeast *Candida albicans* ATCC 10221 and *Penicillium glabrum* (Op694171), were grown on Sabouraud Dextrose Agar (Difco, Lebanon). After inoculation, the seeded plates were held at 4 °C for 1 h to allow for microbial stabilization prior to incubation. Test wells (6–8 mm in diameter) were created on the agar surface using a sterile cork borer, and 100 μL of the plant extract was carefully dispensed into each well. The bacterial culture plates were incubated at 37 °C for 24 h, whereas the fungal culture plates were maintained at 30 °C for 72 h. Antimicrobial activity was assessed by measuring the diameter of the inhibition zone (in mm) surrounding each well. Plates inoculated with microbial strains but without the test extract served as negative controls. Positive controls included gentamicin (1.0 mg/mL) for bacterial strains and fluconazole (1.0 mg/mL) for fungal strains.

### 3.5. MIC Determination of P. ovata Extracts

The minimum inhibitory concentrations (MICs) of *P. ovata* extracts obtained at 40 °C and 80 °C were identified using the broth microdilution method, following the procedure described by Bazaid et al. (2025) [71]. Mueller–Hinton broth was used as the culture medium. Extracts were subjected to two-fold serial dilutions to obtain a concentration range from 0.98 to 1000 μg/mL. Each well of a sterile polystyrene microtiter plate was loaded with 200 μL of the diluted extract mixed with Mueller–Hinton broth. Microbial inoculants were prepared from fresh cultures adjusted to match 0.5 McFarland turbidity standard using sterile Mueller–Hinton broth. A volume of 1–2 μL of the standardized inoculum was added to each well to achieve a final concentration of approximately 3 × 10^6^ CFU/mL. The plates were incubated under microaerophilic conditions (15% CO_2_) at 35 °C for a duration of 72 h. Positive controls consisted of wells inoculated with microbial suspensions but without extracts, while negative controls consisted of wells containing serial dilutions of the extract without microbial inoculation. After incubation, microbial growth was assessed by measuring turbidity at 630 nm using a Biotech 800 TS microplate reader. To determine MIC values, the optical density (OD) of each test well was compared with that of the positive and negative control wells. MIC was defined as the lowest concentration of the extract that produced a visible inhibition of microbial growth. For the test results to be considered valid, observable growth must occur in the positive control wells.

### 3.6. MBC Determination of P. ovata Extracts

The minimum bactericidal concentrations (MBCs) were determined by subculturing 100 μL from each well exhibiting complete inhibition of microbial growth (as identified in the MIC assay) onto Mueller–Hinton agar plates. For comparison, inoculant from the positive growth control wells were also plated. The plates were incubated at 35 °C for 72 h under microaerophilic conditions, following the protocol previously outlined by Anna et al. (2015) [72]. MBC was defined as the lowest concentration of the extract that resulted in no visible colony formation on the agar surface, indicating bactericidal activity.

### 3.7. Assay of Pancreatic Lipase Inhibition

Standard lipase was obtained from Spectrum Diagnostics, and the lipase inhibitory activity of the extracts was assessed using a modified method previously described by Kim et al. (2010) [73], with *p*-nitrophenyl butyrate (PNPB) serving as the substrate. A 0.1 mM potassium phosphate buffer (pH 6.0) and a PNPB solution were prepared using acetonitrile as the solvent, with the final volume adjusted to 10 mL. A standard lipase solution was freshly prepared by dissolving 10 mg of enzyme in 10 mL of the phosphate buffer (1 mg/mL) under gentle stirring immediately prior to use. Serial dilutions of the plant extracts and orlistat (tetrahydrolipstatin, used as a positive control) were prepared in dimethyl sulfoxide (DMSO) to achieve concentrations up to 1000 μg/mL and stored at −20 °C until use. To determine lipase inhibition, varying concentrations of the extracts and orlistat were pre-incubated with lipase in a reaction mixture containing 0.1 mM potassium phosphate buffer (pH 7.2) and 0.1% Tween 80 for 1 h at 30 °C. The enzymatic reaction was initiated by adding 0.1 μL of PNPB, resulting in a final reaction volume of 100 μL. The mixture was then incubated at 30 °C for 5 min. The amount of *p*-nitrophenol released as a product of enzymatic hydrolysis was measured at 405 nm using a Biosystems 310 Plus UV-Vis spectrophotometer. Negative control reactions were conducted in the presence and absence of inhibitors to assess baseline activity. The percentage of lipase inhibition was calculated using the following equation:

$$Lipase\;inhibitory\left(\%\right)=100-\frac{B-b}{A-a}\times 100$$

where B and b are the absorbance of lipases with inhibitors and the negative control in the presence of inhibitors without lipases, respectively. A and a are the absorbance of lipases without inhibitors and the negative control in the absence of inhibitors/lipases, respectively.

### 3.8. Cytotoxicity Test

Cytotoxicity was assessed using the standard MTT assay on cultured cell lines. Cells were seeded at a density of 1 × 10^5^ cells/mL (100 μL per well) into 96-well tissue culture plates and incubated at 37 °C for 24 h to facilitate the formation of a confluent monolayer. Upon monolayer formation, the culture medium was gently aspirated, and the wells were washed twice with sterile washing buffer. Test samples were prepared via two-fold serial dilution in RPMI maintenance medium supplemented with 2% fetal bovine serum. Subsequently, 100 μL of each dilution was added to the designated wells, with three wells left untreated to serve as negative controls [74,75]. The plates were subsequently incubated at 37 °C for 24 h and examined under a microscope for cytotoxic effects, including indicators such as monolayer disruption, cell shrinkage, rounding, degranulation, and detachment [76,77]. Following incubation, 20 μL of MTT solution (5 mg/mL in phosphate-buffered saline, sourced from BIO BASIC CANADA INC., Markham, ON, Canada) was added to each well. The plate was agitated at 150 rpm for 5 min at 37 °C to ensure the uniform distribution of the reagent, then further incubated under controlled conditions (37 °C, 5% CO_2_) for an additional 4 h to allow viable cells to metabolically reduce MTT into formazan crystals. After incubation, the culture medium was carefully removed, and residual fluid was blotted using sterile paper towels. To solubilize the formazan crystals, 200 μL of dimethyl sulfoxide (DMSO) was added to each well, followed by shaking at 150 rpm for 5 min at 37 °C to ensure complete dissolution. Absorbance was then measured at 560 nm with background correction at 620 nm using a microplate reader [78,79]. Cytotoxic effects were evaluated both macroscopically and microscopically. Morphological changes indicative of cytotoxicity included loss of cellular integrity, disruption of the cytoskeleton, and alterations in nuclear architecture. Damaged cells commonly displayed shrinkage resulting from the substantial loss of intracellular proteins and ions, attributable to the compromised membrane permeability of sodium and potassium. Cell death was characterized by nuclear swelling, chromatin condensation, clumping, and reduced nuclear basophilia. Additionally, nuclear condensation followed by nucleolar fragmentation was observed in cells undergoing apoptotic processes.

### 3.9. Statistical Analysis

Statistical analyses were conducted to calculate mean values ± standard deviation (SD) and average values ± standard error (SE) utilizing Microsoft Excel 365 and IBM SPSS Statistics version 25. Data demonstrating normal distribution among treatment groups were analyzed using one-way analysis of variance (ANOVA), followed by Tukey’s post hoc test to identify statistically significant differences between group means. A *p*-value of less than 0.05 was considered indicative of statistical significance.

## 4. Conclusions

In recent years, medicinal plants have gained increasing attention as alternative therapeutic agents due to their high efficacy and lower incidence of side effects compared to conventional pharmaceuticals. Among the major global health concerns, obesity, cancer, and infectious diseases represent significant examples of non-communicable and communicable diseases, both contributing substantially to human morbidity and mortality. This study highlights the therapeutic potential of *P. ovata* leaf extracts obtained via SFE. The extract prepared at 40 °C exhibited superior antimicrobial, anti-obesity, and cytotoxic activities compared to the 80 °C extract, which may be attributed to its higher content of key polyphenols. Notably, it showed the strong inhibition of pathogenic microbes, pancreatic lipase, and colon cancer (Caco-2) cell viability. These findings suggest that *P. ovata* leaf extracts, particularly those obtained at lower SFE temperatures, possess a promising combination of antimicrobial, anti-obesity, and cytotoxic properties, and may serve as a potential natural therapeutic agent for managing infectious diseases, metabolic disorders, and colorectal cancer. Further investigations, including in vivo studies and molecular mechanism elucidation, are warranted to fully validate its clinical applicability.

## Figures and Tables

**Table 1:** Concentrations of indigenous components of *P. ovata* SFE 40 °C and SFE 80 °C versus standard polyphenols.

Components	Polyphenols		*P. ovata* SFE 40 °C		*P. ovata* SFE 80 °C	
	Conc. (µg mL^−1^)	Area (mAU*s)	Conc. (µg mL^−1^)	Area (mAU*s)	Conc. (µg mL^−1^)	Area (mAU*s)
Ellagic acid	70	689.07	168.62	1659.90	290.57	2860.32
Gallic acid	20	273.16	11.69	159.73	2.77	37.86
Chlorogenic acid	50	358.82	18.81	134.97	5.54	39.74
Catechin	75	349.12	0.00	0.00	0.00	0.00
Methyl gallate	15	268.08	0.06	1.15	0.00	0.00
Caffeic acid	20	389.84	123.00	2397.53	76.51	1491.38
Syringic acid	20	340.02	5.65	96.13	1.70	28.97
Rutin	50	334.02	264.90	1769.64	75.80	506.37
Coumaric acid	20	556.49	0.05	1.28	0.13	3.56
Vanillin	20	551.43	8.69	239.60	11.92	328.55
Ferulic acid	20	344.53	1.01	17.39	0.00	0.00
Naringenin	30	325.05	2.07	22.40	1.35	14.57
Kaempferol	20	795.56	0.18	7.35	0.31	12.47
Hesperetin	20	427.11	3.04	64.93	0.00	0.00
Rosmarinic acid	50	514.83	16.10	165.74	9.83	101.19
Daidzein	20	349.30	3.43	59.88	0.11	1.94
Quercetin	40	321.17	1.82	14.59	1.31	10.52
Cinnamic acid	10	515.96	0.19	9.75	0.19	9.80
Total polyphenols	--		(0.18 g GAE kg^−1^ FW)		(0.13 g GAE kg^−1^ FW)	

## Data Availability

Data that supports the findings of this study are available within the article and from the corresponding author upon request.
